# Multi-Toxin Quantitative Analysis of Paralytic Shellfish Toxins and Tetrodotoxins in Bivalve Mollusks with Ultra-Performance Hydrophilic Interaction LC-MS/MS—An In-House Validation Study

**DOI:** 10.3390/toxins12070452

**Published:** 2020-07-13

**Authors:** Fadillah Putri Patria, Heidi Pekar, Aida Zuberovic-Muratovic

**Affiliations:** Science Department, Swedish Food Agency, Box 622, SE-751 26 Uppsala, Sweden; fadillah.patria@gmail.com (F.P.P.); heidi.pekar@slv.se (H.P.)

**Keywords:** marine biotoxins, bivalve mollusks, paralytic shellfish toxins, tetrodotoxin, UP-HILIC-MS/MS

## Abstract

Ultra-performance hydrophilic interaction liquid chromatography tandem mass spectrometry system (UP-HILIC–MS/MS) was used in multi-toxin analysis of paralytic shellfish toxins (PSTs) and tetrodotoxins (TTXs) in sample matrices from bivalve molluscan species commercially produced for human consumption in Sweden. The method validation includes 17 toxins of which GTX6 and two TTX analogues, TTX and 4,9-anhydroTTX, were previously not analyzed together with hydrophilic PSTs. 11-deoxyTTX was monitored qualitatively with a non-certified reference standard. The performance of the method was evaluated for selectivity, repeatability, and linearity by analyzing spiked samples which generated linear calibration curves across the concentration ranges used (R^2^ > 0.99). The in-house reproducibility (RSD) was satisfactory including the LOD and LOQ for both PST and TTX toxins being far below their regulatory action limits. The major advantage of the method is that it allows direct confirmation of the toxin identity and specific toxin quantification using a derivatization-free approach. Unlike the PST-chemical methods used in routine regulatory monitoring until now for food control, the UP-HILIC-MS/MS approach enables the calibration set-up for each of the toxin analogs separately, thereby providing the essential flexibility and specificity in analysis of this challenging group of toxins. The method is suitable to implement in food monitoring for PSTs and TTXs in bivalves, and can serve as a fast and cost-efficient screening method. However, positive samples would, for regulatory reasons still need to be confirmed using the AOAC official method (2005.06).

## 1. Introduction

Marine biotoxins, such as paralytic shellfish toxins (PSTs) and tetrodotoxins (TTXs), accumulate in bivalve mollusks (e.g., mussels and oysters) during their filter feeding in marine environment where other marine species responsible for generation of these toxins are present (phytoplankton [1] or marine bacteria in symbiosis with other organisms [2,3,4,5,6]). Accumulation of these toxins in edible bivalves is important to keep under observation in the area of seafood safety as they belong to the most neurotoxic substances known to induce serious illness [7] with the risk of fatal intoxication effects when ingested in higher concentrations [8,9,10]. There are also reports that PST (saxitoxin, STX) and TTX have been found together in marine organisms including bivalves [7,11] and Finch et al. demonstrated that their toxicities are additive. More than 50 PST and 30 TTX analogues are known to exist worldwide [7,12], of which a certain number of PSTs are included in the European Commission (EC) regulation for marine toxins in bivalve mollusks, that currently do not cover TTX [Regulation (EC) No 853/2004]. During the recent years, the presence of TTX has been reported in different marine organisms, including gastropods and bivalve mollusks, from diverse geographic regions within European waters [3,13,14,15,16]. The changing climate with longer periods of elevated temperatures in combination with the presence of nutrients create the conditions for new events in the marine environment. These changes can be linked to proliferation of phytoplankton (harmful algae blooms, HABs) [17,18,19] resulting in occurrence of previously known toxins at new places as well as occurrence of new analogs and variants of marine biotoxins [20,21,22,23]. Such emerging toxins pose serious hazard for public health and destabilizes the aqua culture of bivalves.

So far, the toxin profile in Swedish marine waters used for cultivation of bivalves for human consumption has been less complex in comparison to the toxin profile common for southern European/Atlantic marine waters [24]. However, during the recent years, changes of the marine environment in the bivalve production areas have been evident and it has become difficult to predict toxin events based on the previous experience. Following the Swedish national official control (OC) monitoring program the general marine biotoxins profile has become extended with new toxins, still within the scope of the EU regulation (Regulation (EC) No 853/2004). Consequently, the task of identifying previously non-occurring toxins in bivalves produced in Sweden has gained an increasing importance. The detection and quantification of PST group of toxins is a formidable analytical challenge well-known among the PST-monitoring laboratories. Except for the reference method, mouse bioassay (MBA, AOAC 959.08) that has been replaced with chemical methods in most of the monitoring laboratories within EU, there are at present three official AOAC alternative methods for analysis of PSTs. These methods are: pre-column oxidation LC-FL (AOAC 2005.06) [25], post-column oxidation LC-FL (AOAC 2011.02) [26], and the receptor binding assay, RBA (AOAC 2011.27) [27]. In the absence of chemical methods that are more straightforward in application, these have served excellently, but with a lot of effort in time, labor, and highly skilled personnel trained to practice the methods and interpret complex chromatograms. Using these methods, the need for caution of false positives/negatives must always be emphasized whether it is due to matrix interferences or cross-reactions due to the lack of specificity of antibodies. Thus, the need for the development of new, LC-mass spectrometry (LC-MS)-based methods to circumvent the above-described difficulties, has been recognized for a long time. During the significant effort in method development to direct the analysis of PST toxins toward MS, the MS methodology faced multiple demands like low detection limits, sample matrix issues, the presence of salts, and co-extraction of undesired compounds [28,29,30,31,32], which has slowed down the application of MS in routine analysis of bivalve samples. Finally, HILIC separation preceded by a reasonable sample preparation procedure was presented by Boundy et al. [33] as a promising approach for routine determination of the highly polar compounds of PST group in shellfish.

Since the application of HILIC-MS/MS has not been widely accepted in routine monitoring analysis, the aim of this study is to investigate its usefulness and robustness in analysis of PSTs and TTXs in the same method, and for their quantitative determination using an UP-HILIC-MS/MS system in our lab. In this report, the performance of the method is presented in an in-house validation study of PSTs and TTXs in sample matrices of blue mussels, oysters, and cockles, with a further aim to apply the method in screening of PSTs and survey of TTXs in bivalves produced in Sweden.

## 2. Results and Discussion

**Figure 1:**
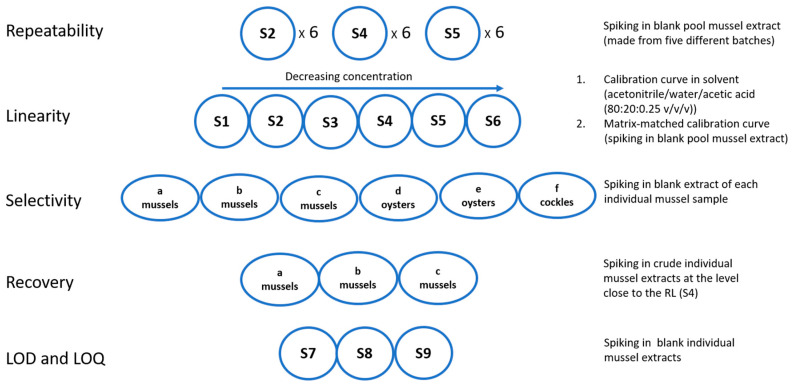
Illustration of the set-up of one single validation experiment. Three of such experiments were performed in a period of 2.5 months including 18 individual bivalve samples from three bivalve species (marked as a–f of total a-r included in the validation). S1–S6, for linearity, indicate calibration standards at six concentration levels covering slightly different concentration ranges for the different toxin analogues. Sum concentration of all PST analogues in S4 corresponds, approximately, to the regulatory limit level (RL) in monitoring of paralytic shellfish toxins (PSTs) in bivalves within the European Union (EU), 800 µg STXeq/kg. For TTX the RL recommended by EFSA, 44 µg TTX/kg, was used as reference, which approximately corresponds to TTX concentration in S5.

The most common bivalve species commercially produced in Sweden, blue mussels (*Mytilus edulis*), oysters (*Ostrea edulis*), and cockles (*Cerastoderma edule*) were used as three representative model samples for our lab. These species naturally occur to varying extent in Swedish west coast waters, which is also in proportion to the share of each of the species in the harvesting for human consumption [34]. The validation scheme in Figure 1 shows the set-up of this study where the contribution of each of the respective species approximately reflects the harvesting pattern of shellfish for which the validated method will be used to monitor. Planning and conducting of the validation experiments included the principles of analysis from the previous works published by Boundy et al. [33] and Turner et al. [35] suggesting that TTX possibly could be analyzed in parallel with PST, although different types of adjustments and preferences that were more suitable for our lab were made (see Section 4).

The experiments shown in the validation scheme were repeated at three different time points within a period of 2.5 months (from the beginning of March to mid of May with approximately 3 and 4 weeks between the experiments). The evaluated parameters were selectivity, linearity, repeatability, recovery in sample cleaning, limit of quantification (LOQ) and limit of detection (LOD). The method was also used for quantification of the PST analogues in the proficiency test administrated by the European Union Reference Laboratory for Marine Biotoxins (EURL-MB) in 2019. During 2017–2018, the method was used to identify the PST analogues that in a second step were quantified using AOAC 2005.06. The extraction step using 1% acetic acid in analysis of PST is an extensively used and established procedure in regulatory monitoring of PST toxins in bivalves [36,37] as well as the extraction of TTX using 1% acetic acid was later included in the initial HILIC-MS analysis of this toxin [15,35,38]. Investigation of variations in the extraction efficiency was thus considered to be beyond the scope of this report. More attention was devoted to the clean-up step of the sample extracts known to be critical in analysis of hydrophilic toxins in bivalves with LC-MS as the presence of salts and co-extractives causes interferences that significantly influence chromatography and lead to suppression in electrospray ionization. Upstream in the analysis using UP-HILIC-MS/MS a proper start-equilibration of the system before each sample batch is, according to our experience, an inevitable prerequisite crucial for a successful, repeatable qualitative analysis and determination of PST and TTX toxins. This routine also refers to a proper system equilibration through multiple injections of extract from blank matrix before samples with the corresponding matrix are injected. Of a similar importance for the repeatability of analysis is also the composition of solvent versus water in the samples (approximately 80% acetonitrile throughout this study). Once these requirements were fulfilled, the analysis using UP-HILIC-MS/MS ensured good performance of the method for all PST and TTX analogues included in this report. Although specific retention time window groups could be chosen for each of the ionization modes (ESI+ and ESI−) in multiple reaction monitoring (MRM) method, the detection time window for all groups was set to 1.5–11 min. This to anticipate possible instability of the retention times of the toxins, which would be difficult if they were set in narrower detection time window. However, during the entire validation study no retention time drifts were observed for any of the toxin analogues. For some of the toxins no reference standard was available, still the MRM transitions of these toxins were of interest to keep in the method for monitoring since they are contributing to the toxicity of the sample.

### 2.1. Specificity

**Figure 2:**
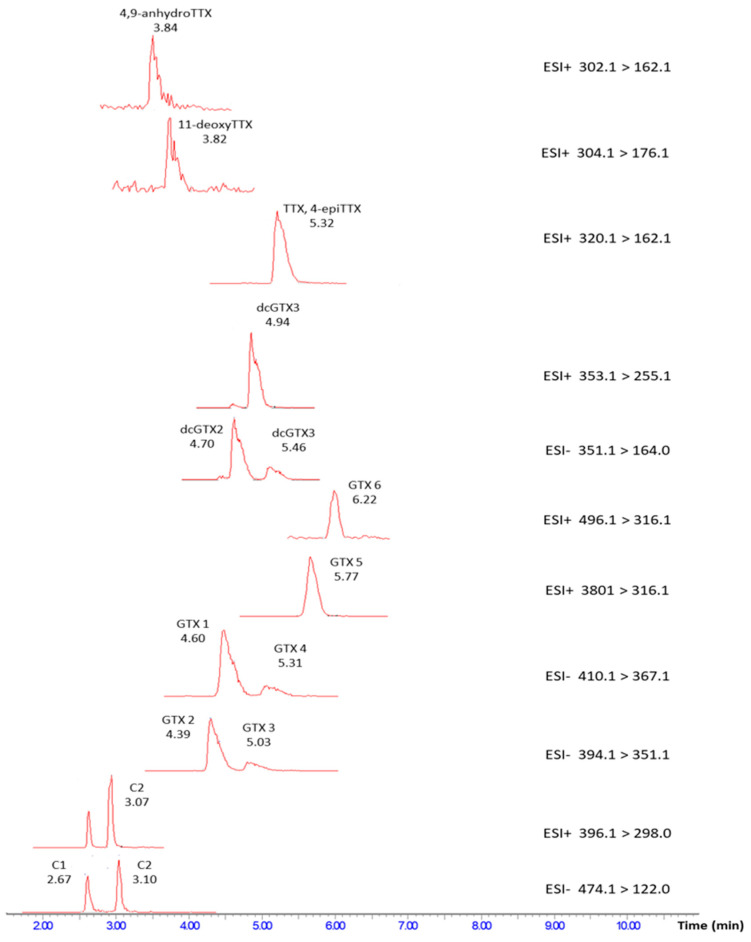
MRM transitions of TTX analogues and some of the PST analogues critical in selectivity evaluation, spiked at mid-level concentrations (S4) in matrix extract from blue mussel. No difference in selectivity was observed between different species tested, blue mussel, oyster, and cockles. (MRM chromatograms of other toxins can be found in Supplementary Material Figure S2).

To investigate the specificity of the method, individual shellfish samples were used each corresponding to a homogenate from a batch of mussels (1 kg) harvested at a specific location and labeled with a lot number indicating a specific harvesting time. Nine of such different supposedly blank blue mussel samples, six of oysters, and three of cockles (18 in total) were analyzed over three different days with respect to the presence of peaks that could disturb or be misidentified as the peaks of the PST or TTX analogues. No interferences were found at concentration levels ≥LOD in any of these samples (blank sample chromatograms are not shown). The use of both electrospray ionization modes (ESI+ and ESI−) is an approach that increases the specificity of the method for many toxins, as well as it, according to our experience, further simplifies the evaluation of an analysis if applied as separate injections. This benefits the confirmation of the identification for GTX3-6, dcGTX3, and C1&2 toxins that give MRM transitions both in ESI+ and ESI− mode. The precursor ions of GTX6 and C1 are the same, yet the product ions and the retention times of these toxins are different. On the other hand, GTX2&3, GTX1&4, and C1&2 are acquired with the same MRM transitions, which applies the same rule in the identification of each of these toxins that is based on their retention time differences (Figure 2). Furthermore, different quantification product ions were employed for the quantification of the epimers with the same MRM transitions, e.g., GTX1&4 and GTX2&3. According to the presented results, the method is considered as specific for PST and TTX toxins included in this report.

### 2.2. Calibration and Linearity

Two series of calibration standard solutions were analyzed (in solvent and in matrix-matched solution) on three different days to evaluate the linearity of each of the toxins. The calibration standard solutions were prepared at six concentration levels in each of the calibration series. The calibration range was chosen by placing the total regulatory-limit-concentration of 800 µg STXeq/kg (in which all the PST toxins were represented) approximately in the middle of the calibration range, S4, and the corresponding level for TTX was at S5 (Figure 2). The set-up resulted in slightly different calibration ranges for different toxin analogues (Table 1). This model could reflect a naturally dynamic profile of PSTs and TTXs content in a complex shellfish sample where the toxins might exist in varying concentrations. Prior to analysis the toxin stock solutions were kept separately because of the different storage conditions requirements for each of the toxins (−20 °C or +4 °C) and to avoid the risk of possible transformation of the analogues when stored in a mixture. Recalling the variation of the toxin composition that might exist in a natural sample, the separate storage of stock solutions also implies the cost and flexibility benefits when the preparation of a calibration curve need to be adapted to a simpler toxin profile of a sample. The derived calibration curves were linear in the tested calibration range for both calibration series (in solvent and matrix-matched). The slopes of the two-calibration series were used according to Equation (1) to assess matrix-related signal effects in the electrospray ionization indicating suppression or enhancement of the signal if below or above 100%, respectively. The results of the linear regression and the matrix effects are presented in Table 1 and demonstrate the importance of using matrix-matched calibration curve in quantitative analysis of toxins in authentic samples. To visualize the matrix effects the calibration curves of each toxin prepared in solvent and in matrix are shown in Supplementary Material Figure S1.

(1) $$\%ME=\frac{Slope\;of\;the\;Calibration\;Curve\;in\;Matrix\;}{Slope\;of\;the\;Calibration\;Curve\;in\;Solvent\;}\;\times 100$$

### 2.3. Recovery in Sample Preparation and Matrix Effects in ESI-MS

Recoveries of PST and TTX were determined through analysis of nine individual blue mussels extracts spiked prior to SPE clean-up. The approach to spike crude extracts was based on the cost-related calculations of the reference standard amounts needed to spike the homogenate tissues for the whole validation study. Furthermore, this first step in the extraction procedure is well tested through a long experience of use in reference methods, like HPLC-FL (AOAC 2005.06), and the proficiency tests organized by EURLMB (European union reference laboratory for marine biotoxins). Evaluation of the recovery in PSTs analysis for HILIC-MS/MS was thoroughly carried out by Turner et al. [39] using at that time available certified reference material (CRM). Investigation of the recovery efficiency from homogenates was thus considered to be beyond the scope of this work. The toxin concentrations in spiked sample extracts for recovery study were chosen to be equal to S4 (Figure 1), approximately corresponding the total regulatory limit (RL) for PST when all toxins are present in the sample, while for TTX it corresponded to three times its RL (44 µg/kg) [3]. The extracts were cleaned up with SPE, diluted and analyzed by UP-HILIC-MS/MS on three separate days. The recovery values presented in Table 2 were calculated from peak areas of measured values and the spiked (nominal) values. Most individual toxin recoveries ranged from 54 to 74%, although recoveries were notably lower for GTX6 (34%) and C1 (49%). Lower recoveries were expected for C1 due to the presence of sodium formate salt, which is present at low concentrations in solution even after carbon SPE clean-up, as also reported by Boundy et al. [33]. In previous studies, the recovery of GTX6 has not been presented in analysis of PSTs in bivalves due to unavailability of certified reference standard for this toxin. In this work, the recovery of GTX6 was lower than expected. The values obtained so far will be observed, and the measures to increase the recovery will be conducted continuously to improve the performance in GTX6 analysis. In general, the values of relative standard deviations in repeatability of recovery within- and between-batches showed in Table 2, are in agreement with the established guidelines.

### 2.4. Quantitative Analysis

The total toxicity of PST was expressed as µg STX equivalent per kg of sample (µg STXeq/kg) using the toxicity equivalency factor (TEF) values of each PST analogue recommended by EFSA [40]. Since the TEF values for C1 and TTX analogs were not available in EFSA recommendations the TEF for C1 was adopted from a previous publication [33] while the total concentration of TTX was expressed as µg TTX/kg without considering TEF factor. One of the TTX analogues, 11-deoxyTTX, was present in the certified reference standard solution of TTX at a concentration that was not certified. The analysis of 11-deoxyTTX was therefore kept in the validation study for the purpose to evaluate the qualitative performance of the method. The quantity of 11-deoxyTTX in this report could be used as a semi-quantitative value. The Equation (2) was used to calculate the concentration found in unknown samples and calibration standards from nmol/L to µg STXeq/kg.

(2) $$Conc\;(\frac{µg\;STXeq}{kg})=\frac{Conc\;\left(\frac{nmol}{L}\right)\times d\times Vol\;\left(L\right)\times MW\;\left(\frac{g}{mol}\right)\times TEF\;}{m\;\left(g\right)}$$

where:

Conc (µg STXeq/kg): concentration of the toxin in the homogenate sample, µg STX eq/kg
Conc (nmol/L): concentration of the toxin found in the sample solution, nmol/L
d: dilution factor throughout the whole extraction procedure in ready-to-inject sample (20)
Vol: total volume of the extract, L (5 mL of 1% acetic acid)
MW: molecular weight of toxin, g/mol
TEF: toxicity equivalency factor of toxin. For C1 a TEF of 0.01 was applied [33]
m: mass of the sample homogenate, g (5 ± 0.1 g)

### 2.5. Repeatibilty

The repeatability was evaluated by spiking SPE-cleaned pool extracts in six replicates at three different concentrations (high, mid, and low) each time of analysis. Within-batch repeatability and between-batch repeatability of each toxin was evaluated for the three concentrations. Within-batch repeatability was calculated as relative standard deviation over six replicates (*n* = 6) for each concentration level and between-batch repeatability (intermediate precision) was calculated as relative standard deviation of three different batches of analysis performed at different time points in a period of 2.5 months (*n* = 18 for each of the three concentrations). Both within- and between-batch repeatability values presented in Table 3 show consistency between the three assessed concentrations for most of the toxins. There was no evidence on concentration-related dependence in the repeatability nor in the matrix effects. The low measurement uncertainty for the quantitative determination in the low concentration range ensures that the analysis of samples containing toxins at low concentrations is not compromised. Both the within- and between-batch repeatability values (RSD_r_ and RSD_R_) are in agreement with the EU validation guideline [41].

### 2.6. LOD and LOQ

LOD and LOQ for PSTs and TTXs were determined in individual low-level spiked mussel sample extracts in nine replicates over three batches of analysis. The three low-level spiked concentrations were below the lowest point in the calibration curve, as illustrated in Figure 1 as S7, S8 and S9. The criteria for signal-to-noise ratio (S/N) was set to ≥3 for the evaluation of LOD and ≥10 for LOQ. However, the analysis of S7–S9 samples showed that the S/N for most of the toxins was above 10, indicating high sensitivity of the method at concentrations far below the RL, both for PSTs and TTXs groups. The results presented in Table 4 show that the lowest LOD was found to be 0.1 µg STXeq/kg for C1. In the evaluation of LOQ for 11-deoxyTTX, 4,9-anhydroTTX and GTX 4 it was found to be higher than the lowest calibration solution, S6. Thus, the LOQ of 11-deoxyTTX, 4,9-anhydroTTX and GTX 4 were 1.89, 7.45, and 12.96 µgTTX/kg and µg STXeq/kg, respectively, which corresponds to calibration solution S5.

## 3. Conclusions

There is an important need to have robust and accurate TTX detection method for screening of shellfish for this potentially fatal toxin that has been emerging on several geographical places within the EU during the recent years. The TTX was thus included in a multi-toxin UP-HILIC-MS/MS method together with the PST group of toxins and, with the support of the previously published thorough method development for PST using HILIC, here presented as a tool qualified to include in the official monitoring of bivalves in the European food control. This report also shows that the progress in using the UP-HILIC-MS for PST and TTX analysis is not only specific for the most experienced labs, but also feasible for the official laboratories performing food control in the member states of EU. The direct analysis of PST and TTX provided high specificity and robustness in simultaneous identification of these groups of toxins at low levels, µg/kg for TTX and µg STXeq/kg for PSTs, in our lab. The flexibility of the used approach offers the possibility to extend the analytical range to other hydrophilic toxin analogues and other bivalve matrices without compromising the qualitative performance of the method. Finally, as the complexity of toxin profile in bivalves is likely to increase in EU because of climate change [42], it is necessary to have a method that can meet the analytical needs without doubtfulness in toxin identification and complex calculations in the determination of sample toxicity, as it is when using the official HPLC-FLD-based PST methods. Lastly, in some countries, the bivalve industry is struggling with profitability and because of the toxic nature of the marine biotoxins there are extensive but also costly control programs in place within EU. The proposed method is also cost efficient, since two groups of marine biotoxins can be monitored simultaneously.

## 4. Materials and Methods

### 4.1. Standards and Reagents

Certified reference standards of toxins STX, gonyautoxins 1-6 (GTX1-6), neosaxitoxin (NEO), decarbamoylsaxitoxin (dcSTX), dicarbamoyl neosaxitoxin (dcNEO), N-sulfocarbamoyl gonyautoxin-1&2 (C1 and C2), and decarbamoylgonyautoxin-2&3 (dcGTX2 and dcGTX3) were obtained from the Institute of Biotoxin Metrology, National Research Council Canada (NRCC, Halifax, NS, Canada). Certified reference standard of TTX and the analogues were obtained from Laboratorio CIFGA (Lugo, Spain). The summary of toxins and their concentrations is presented in Table 5. Solvents used for mobile phase preparation and all other chemicals were of LC-MS grade where possible, acetonitrile (ACN, Fisher Scientific, Loughborough, UK), methanol, and formic acid 98–100% (Merck, Darmstadt, Germany). Amorphous graphitized polymer carbon Supelco ENVI-Carb 250 mg/3 mL cartridges (Sigma-Aldrich, St. Louis, MO, USA) were used. Sample preparation and SPE reagents were of HPLC grade.

### 4.2. Materials

Fresh blue mussels (*Mytilus edilus*), oysters (*Crassostrea gigas*), and cockles (*Cerastoderma edule*) were purchased from local stores in Sweden to use as blank samples. For sample preparation a Grant - SBB Aqua 5 Plus boiling water bath, a Vortex Genie 2 Scientific Industries, and a Thermo Scientific Haraeus Multifuge 3SR+ centrifuge were used. Solid phase extraction (SPE) was automated on a Gilson Aspec GX-274 instrument with 3 mL SPE racks (code 304) and an 80 positions tube rack (code 330). Mass spectral experiments were performed using Waters Xevo TQ-S triple quadrupole mass spectrometer coupled to a Waters Acquity UPLC i-Class with a flow through needle sample manager.

### 4.3. Sample Preparation

#### 4.3.1. Homogenization of Shellfish Samples

In this study the samples were purchased from a local grocery store as packages of 1 kg fresh mussels/oysters/cockles. One such package corresponded to one sample batch and all samples purchased were from different lot numbers indicating different harvesting time. In general, at least 15 whole individuals without shell were used for one sample (approx. 50–200 g). The outside of the shellfish was cleaned with tap water. Shellfish was opened by cutting adductor muscle and the tissue was removed from shells by separating adductor muscles and the tissue connecting at the hinge. The shelled shellfish was drained for 5 min in a sieve. A grinder with 2500 rpm was used for 1.30 min to homogenize the shellfish tissue, and then the homogenized tissue was weighed in portions and were either directly used for the extraction or could be stored in −20 °C until needed. A pool of blank blue mussel samples was prepared by mixing the homogenates of mussels from five different harvesting batches. The extract from the blank pool sample was used to prepare calibration solutions. 

#### 4.3.2. Shellfish Extraction and Clean Up

Shellfish extraction and clean-up were performed as described by Boundy et al. [33]. Briefly, the homogenates of samples were extracted using a single dispersive extraction procedure: 5 ± 0.1 g of shellfish homogenate was weighed in a 50 mL centrifuge tube and 5 mL of acetic acid/water (1:100, *v*/*v*) was added. The sample tubes were shaken and loosely sealed and placed in a boiling water bath for 5 min, and then they were cooled to room temperature. Samples were remixed on a vortex mixer, for 5 min to extract TTXs while the PSTs were extracted during the initial 90 s of mixing, and then centrifuged at 3600× *g* for 10 min. The supernatant was transferred to a clean sample tube and a portion of the supernatant was proceeded to SPE clean up. An aliquot of 1 mL of the acetic acid extract was transferred to a polypropylene tube and 5 µL of NH_4_OH was added. The SPE procedure was performed on a SPE liquid handling robot (Aspec GX-274) with amorphous graphitized polymer carbon cartridges (Supelco, Supelclean^TM^ ENVI-carb 250 mg/3 mL). Total of 3 mL of acetonitrile/water/acetic acid (20:80:1, *v*/*v*/*v*) was applied with 200 µL air push to condition the SPE column, followed by 3 mL of water/NH_4_OH (1000:1(25% NH_4_OH), *v*/*v*) with a 200 µL air push. Total of 400 µL of sample extracts were loaded into the conditioned SPE column with a 200 µL air push, thereafter the columns were washed with 700 µL of deionized water applying a 400 µL air push to elute to waste. Clean SPE-retentate of the sample was then eluted into a polypropylene tube with 2 mL of acetonitrile/water/acetic acid (20:80:1, *v*/*v*/*v*) and a 400 µL air push. For the UP-HILIC-MS/MS analysis an aliquot of 100 µL of the SPE eluent was mixed and diluted by adding 300 µL of acetonitrile in an autosampler vial and a 5 µL injection volume of this mixture was used in each run.

### 4.4. Calibration Curve

Separate stock solutions of each toxin were prepared by accurately pipetting 100 µL of reference standard from toxin ampule to 900 µL of water, to give a final volume of 1.0 mL. The stock solutions were stored refrigerated or frozen according to the instruction for storage of each specific toxin. These separate stock solutions were used to prepare fresh LC-MS/MS calibration solutions at each different day of analysis, for spiking of samples to evaluate repeatability, selectivity, recovery, and for the determination of LOD and LOQ of the method. Calibration solutions in solvent were prepared at six concentration levels different for each of the toxins by diluting the mixed stock solution into a diluent of 80% acetonitrile (MeCN) with 0.25% acetic acid, while calibration solutions in matrix were prepared in SPE-cleaned blue mussel pool sample extract (for more information see Supplementary Material Table S1).

### 4.5. UP-HILIC-MS/MS

UP-HILIC-MS analysis was performed using a 1.7 µm, 150 × 2.1 mm Acquity UPLC BEH Amide column with a VanGuard BEH Amide guard column and Waters UPLC I-Class (Waters, Milford, MA, USA) with Waters Xevo TQ-S mass spectrometer system (Waters, Milford, MA, USA). The column was held at 60 °C and the autosampler at 15 °C. Mobile phases were as follows; A1: water/formic acid/NH4OH (500:0.075:0.3, *v*/*v*/*v*); B1: acetonitrile/water/formic acid (700:300:0.1, *v*/*v*/*v*); A2: water/acetonitrile (95:5, *v*/*v*); B2: water/acetonitrile (5:95, *v*/*v*). The sample injection volume was 5 µL. The ionization parameters were as follows: capillary voltage 3.0 kV, source desolvation temperature 600 °C, and source ion block temperature of 150 °C. Nitrogen (≥95%) desolvation gas flow rate was set at 1000 L/h, and nebulizer gas at 7.00 Bar. In addition, Argon (zero grade) collision gas flow was set at 0.15 mL/min. At the start of each batch a pre-conditioning of the column was performed. The column was initially flushed with 50:50 A1:B1 at 0.2 mL/min for 30 min where after the flow rate was gradually increased over 5 min to 0.4 mL/min and a gradient was started 5:95 A1:B1 over next 3 min. This condition was held for 2 min and corresponded to the solvent composition at the initial part of the chromatographic gradient. The pre-conditioning was automated and the first injection of the sample batch started immediately after the column pre-conditioning. The chromatographic gradient conditions from Boundy et al. were slightly modified and optimized for use in our lab. The initial chromatography conditions 5:95 A1:B1 were run for 4 min, then a linear gradient started to 50:50 A1:B1 over 3.5 min. This composition was held over 1.5 min. The column then underwent a linear gradient to 95:5 A1:B1 over 0.3 min and was held for 0.5 min. Followed by re-equilibration to 5:95 for 0.8 min and then held for 0.4 min. Total acquisition time of the elution was 11 min. The flow rate was 0.4 mL/min throughout the entire gradient elution. At the end of each sample batch the column was washed with 100:0 A2:B2 for 5 min at 0.2 mL/min, followed by a linear gradient 0:100 A2:B2 over 5 min. The flow was then linearly increased to 0.4 mL/min over 2 min. The composition and the flowrate was then held for 33 min and the column was stored in B2 (water/acetonitrile (5:95, *v*/*v*)). Both positive (ESI+) and negative (ESI−) electrospray ionization and multiple reaction monitoring (MRM) were used for UP-HILIC-MS/MS analysis of PST and TTX. MS/MS conditions earlier reported for PST and TTX [33] and the specific product ions that were produced from the selected precursor ions were used in our lab for available standards across the mass range m/z 40-600. A minimum of two transitions were used for each PST and TTX analogue for the MRM analysis (Table 6 and Table 7), either in one ionization mode or in both ionization modes together (could be one in positive and one in negative ESI, e.g., for GTX toxins, dcGTX toxins and C toxins). In this study separate injections were done in positive and negative ESI mode. Two MRM methods were built separately. In each MRM method functions were grouped according to the similarity in the chromatographic retention times of toxins. Selected precursor ions in MS1 and their corresponding fragment ions in MS2 are presented in Table 6 and Table 7 with the settings of the MS and MS/MS analysis summarized in detail. The bold m/z values indicate the MRM transitions with the highest abundance in MS2 used for quantification of corresponding analogues. For quantitative analysis a specific calibration curve was built for each of the toxin analogues using Targetlynx v 4.1 software (Waters, Milford, MA, USA, 2011). The extracts from the three bivalve species (blue mussels, oysters, and cockles) prepared according to the procedure described in Section 4.3, and spiked with a standard mixture of PST and TTX toxins at different concentration ranges for different toxin analogues (Supplementary Material Figure S1 and Table S1), were injected to obtain calibration curves. These were constructed by plotting peak area of the toxin analogue against concentration of the standard using linear regression. Each time of analysis corresponding calibration curves were also prepared in solvent. The calibration curves prepared in solvent and in matrix were injected according to the bracketing principle the analysis of a sample batch, which provided a reliable base for the control of the retention times for target toxin analogues.

## Figures and Tables

**Table 1:** Linear regression parameters of solvent and matrix-matched calibration curves for seventeen toxin analogues. Matrix effect was evaluated as %ME calculated according to Equation (1) for each toxin: %ME = 100—no signal effect, %ME < 100—signal suppression, %ME > 100—signal enhancement. Italic values represent calibration range for TTX. Calibration curves of each toxin that visualize the signal suppression/enhancement can be found in Supplementary Material Figure S1.

Table	Calibration Range		Correlation Coefficient (*R*^2^)		Matrix Effect (ME)
	nmol/L	µg STX eq/kg *µgTTX/kg*	Solvent	Matrix-Match	%ME
dcSTX	4.1–130.0	21.0–671.5	0.995	0.998	59
dcNEO	1.9–60.8	5.25–167.9	0.991	0.995	69
STX	4.1–132.6	24.8–793.7	0,996	0.997	65
NEO	4.1–130.2	25.7–821.2	0.991	0.996	81
TTX, 4-epiTTX	4.9–157.2	*31.4*–*1003.3*	*0.993*	*0.999*	*45*
11-deoxyTTX	0.2–5.0	*0.9*–*30.3*	*0.997*	*0.998*	*182*
4,9-anhydroTTX	0.6–19.8	*3.4*–*119.2*	*0.996*	*0.998*	*169*
dcGTX3	0.7–21.6	3.8–120.1	0.998	0.999	75
GTX6	2.0–64.5	0.6–19.8	0.996	0.997	76
GTX5	6.2–200.2	2.8–88.8	0.998	0.999	86
C2	1.8–58.8	2.0–64.5	0.997	0.999	59
GTX2	3.6–114.4	20.2–649.0	0.998	1.000	98
GTX3	1.1–36.0	12.9–412.7	0.998	0.999	81
GTX1	6.4–205.2	29.4–942.2	0.997	0.999	102
GTX4	2.7–87.0	6.5–207.3	0.990	0.998	93
dcGTX2	3.7–117.0	20.3–649.0	0.996	0.999	73
C1	0.8–25.0	0.67–21.6	0.999	0.999	51

**Table 2:** Recovery values in sample preparation for spiked crude extracts of individual blue mussel samples, calculated from peak areas. Spiked concentrations in nmol/L and corresponding in µg STXeq/kg of each toxin and the recovery percentages for within- and between-batch repeatabilities. RSD_r_: within-batch repeatability. RSD_R_: between-batch repeatability. All values are based on three repetitions and nine injections. Italic values represent TTX.

Toxins	Conc. in Spiked Sample		Mean Recovery (*n* = 9) (%)	RSD_r_ (%)	RSD_R_ (%)
	nmol/L	µg STX eq/kg *µg TTX/kg*			
dcSTX	16.25	83.94	66	7	18
dcNEO	7.60	20.99	62	10	22
STX	16.58	99.21	65	5	15
NEO	16.28	102.63	65	8	19
TTX, 4-epiTTX	19.65	*125.41*	*68*	*20*	*21*
11-deoxyTTX	0.63	*3.79*	*71*	*18*	*18*
4,9-anhydroTTX	2.48	*14.90*	*72*	*12*	*13*
dcGTX3	7.35	15.01	68	6	14
GTX6	14.62	2.47	34	24	52
GTX5	3.13	11.09	61	7	18
C2	8.48	8.06	54	20	23
GTX2	25.65	81.13	62	19	24
GTX3	10.88	51.59	66	11	16
GTX1	14.30	117.64	64	18	25
GTX4	4.50	25.91	74	12	30
dcGTX2	25.03	34.07	70	10	16
C1	28.35	2.70	49	30	31

**Table 3:** Summary of the repeatability evaluation. Table shows values for within-batch repeatability (RSD_r_) and between-batch repeatability (RSD_R_) for each PST and TTX analogue at high, mid and low concentration in pool extracts of blue mussels. * Concentrations for TTXs are expressed in µg TTX/kg.

Toxins	High-Level			Mid-Level				Low-Level		
	Conc (µg STX eq/kg) *	%RSD_r_ (*n* = 6)	%RSD_R_ (*n* = 18)	Conc (µg STX eq/kg) *	%RSD_r_ (*n* = 6)	%RSD_R_ (*n* = 18)	Conc (µg STX eq/kg) *		%RSD_r_ (*n* = 6)	%RSD_R_ (*n* = 18)
**dcSTX**	335.66	2	9	86.06	3	5	43.44		4	11
**dcNEO**	84.31	3	8	21.98	6	7	11.93		6	10
**STX**	395.35	4	7	102.47	2	4	53.87		2	10
**NEO**	411.77	3	10	105.75	4	6	54.29		4	9
**TTX, 4-epiTTX**	501.89	2	7	124.71	3	6	64.94		4	5
**11-deoxyTTX**	14.56	4	9	3.82	8	14	2.00		9	12
**4,9-anhydroTTX**	55.01	3	12	14.22	12	13	7.41		10	16
**dcGTX3**	74.82	2	3	19.52	3	3	10.65		3	3
**GTX6**	9.91	3	10	2.53	5	12	1.25		10	15
**GTX5**	43.70	1	6	11.15	2	7	5.88		3	9
**C2**	29.85	3	15	7.86	5	13	4.26		8	9
**GTX2**	313.05	5	8	81.00	4	7	40.55		5	7
**GTX3**	198.16	6	8	52.38	6	6	27.90		7	9
**GTX1**	470.33	5	6	117.73	5	4	58.58		6	8
**GTX4**	105.04	8	11	27.93	9	10	14.22		15	18
**dcGTX2**	133.61	3	5	34.21	5	6	17.28		6	6
**C1**	9.99	3	5	2.67	5	10	1.57		6	18

**Table 4:** LOQ and LOD level for each PST and TTX. * These values correspond to a higher LOQ, which is calibration solution S5.

Toxins	LOQ Conc	LOD Conc
	(µg STX eq/kg or µg TTX/kg)	
dcSTX	20.99	2.62
dcNEO	5.25	2.62
STX	24.80	3.10
NEO	25.66	6.41
TTX, 4-epiTTX	15.68	7.84
11-deoxyTTX	1.89 *	0.95
4,9-anhydroTTX	7.45 *	3.73
dcGTX3	3.75	0.94
GTX6	0.62	0.31
GTX5	2.77	0.35
C2	2.01	0.50
GTX2	20.28	2.54
GTX3	12.90	6.45
GTX1	29.41	14.71
GTX4	12.96 *	6.48
dcGTX2	8.52	4.26
C1	0.67	0.08

**Table 5:** Information of the certified reference standard for each toxin and the concentrations. * non-certified reference value.

Certified Reference Standard	Containing Toxins	Concentration (µmol/L)	Commercial Provider
CRM-dcGTX2&3-c	dcGTX2	100.1	NRCC, NS, Canada
	dcGTX3	29.4	
CRM-dcSTX-b	dcSTX	65	
CRM-GTX1&4-d	GTX1	57.2	
	GTX4	18	
CRM-GTX2&3-d	GTX2	102.6	
	GTX3	43.5	
CRM-NEO-d	NEO	65.1	
CRM-STX-f	STX	66.3	
CRM-C1&2-b	C1	113.4	
	C2	33.9	
CRM-dcNEO-d	dcNEO	30.4	
CRM-GTX5-c	GTX5	55.7	
CRM-GTX6	GTX6	12.5	
	GTX5	2.79	
CRM-03-TTXs	TTX	78.6	CIFGA; Lugo, Spain
	4,9-anhydro TTXs	9.9	
	11-deoxyTTX *	2.50	

**Table 6:** MRM transitions in positive ESI mode. The MRM method was built by grouping of toxins with similar retention times in chromatographic separation. Bold ions indicate primary MRM transition for quantification of corresponding analogues. * Toxins for which no primary certified reference standard was available on the market.

Time Windows Group	Analytes	Precursor Ion, *m*/*z*	Product Ion, *m*/*z*	Cone, V	Dwell, ms	CE, eV
1	* dido-dcSTX	**225.1**	**166.1**; 60	10	40	15
2	STX	**300.1**	**204.1**; 138	10	40	24; 30
	NEO	**316.1**	298.1; 220.1; **126.1**	10	40	15; 24; 26
	dcSTX	**257.1**	222; **126.1**	10	40	22; 19
	dcNEO	**273.1**	225.1; **126.1**	10	40	18; 20
	* doSTX	**241.1**	206.1; **60**	10	40	22; 23
3	TTX, 4-epi TTX	**320.1**	**162.1**; 302.1	40	25	38; 25
	11-deoxy TTX	**304.1**	176.1; 286.1	40	25	30
	4,9-Anhydro TTX	**302.1**	162.1; 256.1	40	25	30
	* 11-nor TTX-6-ol	290.1	162.1; 272.1	40	25	30
	* 5,6,11-Trideoxy TTX	272.1	162.1; 254.1	40	25	30
4	GTX3	396.1	298.1	10	50	17
	GTX4	412.1	314.1	10	50	18
	dcGTX3	**353.1**	**255.1**	10	50	18
	* dcGTX4	**369.1**	**271.1**	10	50	18
5	GTX5	**380.1**	**300.1**	10	100	16
	GTX6	**396.1**	**316.1**	10	100	15
6	C1	396.1	298.1	18	40	20
	C2	**396.1**	**298.1**	18	40	20
	* C3	412.1	332.1; 314.1	18	40	16; 20
	* C4	**412.1**	332.1; **314.1**	18	40	16; 20

**Table 7:** MRM transitions in negative ESI mode. The MRM method was built by grouping of toxins with similar retention times in chromatographic separation. Bold ions indicate primary MRM transition for quantification of corresponding analogues.

Time Windows Group	Analytes	Precursor Ion, *m*/*z*	Product Ion, *m*/*z*	Cone, V	Dwell, ms	CE, eV
1	GTX2	**394.1**	**351.1**; 333.1	10	60	16, 22
	GTX3	**394.1**	351.1; **333.1**	10	60	16; 22
	GTX1	**410.1**	**367.1**; 349.1	10	60	15; 22
	GTX4	**410.1**	367.1; **349.1**	10	60	15; 22
	dcGTX2	**351.1**	333.1; **164.0**	10	60	17; 30
	dcGTX3	351.1	333.1; 164.0	10	60	17; 30
2	GTX5	378.1	122	10	100	25
	GTX6	394.1	122	10	100	25
3	C1	**474.1**	351.1; **122.0**	10	40	25; 30
	C2	474.1	351.1; 122.0	10	40	25; 30

## Supplementary Materials

The following are available online at https://www.mdpi.com/2072-6651/12/7/452/s1, Figure S1. Comparison of the slopes in matrix matched calibration curves and the calibration curves in solvent for each of the toxins, showing matrix effects as distinct signal suppression for STX, NEO, dcSTX, dcNEO, GTX3, GTX6, dcGTX2, dcGTX3, C1, C2 and TTX; low signal suppression for GTX2, GTX4 and GTX5; signal enhancement for 4,9-anhydroTTX, 11-anhydroTTX and no matrix effect for GTX1, Figure S2. Full MRM transitions of each toxins at mid-level concentration spiked in blue mussel matrix, Table S1. Concentrations of the calibration standard solutions.
