# Supplementary material for: Multi-Toxin Quantitative Analysis of Paralytic Shellfish Toxins and Tetrodotoxins in Bivalve Mollusks with Ultra-Performance Hydrophilic Interaction LC-MS/MS—An In-House Validation Study
Source: Toxins (Basel). 2020 Jul 13;12(7):452. doi: 10.3390/toxins12070452 (PMC7404990; doi:10.3390/toxins12070452)
Supplement: Supplementary file 1 [file toxins-12-00452-s001.pdf]

Supplementary Material: Multi-Toxin Quantitative Analysis of Paralytic Shellfish Toxins and Tetrodotoxins in Bivalve Mollusks with Ultra-Performance Hydrophilic Interaction LC-MS/MS—An In-House Validation Study

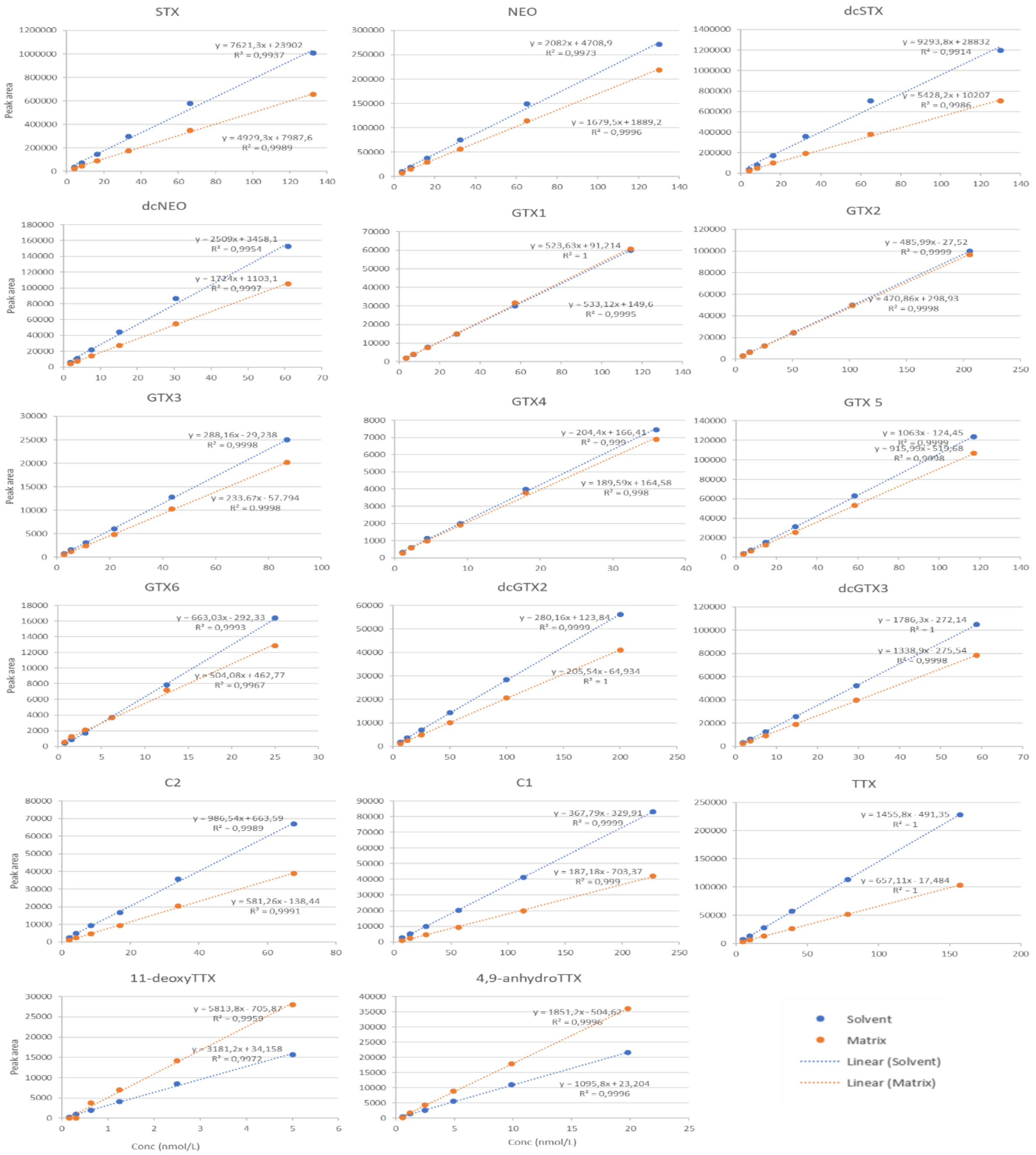

Figure S1. Comparison of the slopes in matrix matched calibration curves and the calibration curves in solvent for each of the toxins, showing matrix effects as distinct signal suppression for STX, NEO, dcSTX, dcNEO, GTX3, GTX6, dcGTX2, dcGTX3, C1, C2 and TTX; low signal suppression for GTX2, GTX4 and GTX5; signal enhancement for 4,9-anhydroTTX, 11-anhydroTTX and no matrix effect for GTX1.

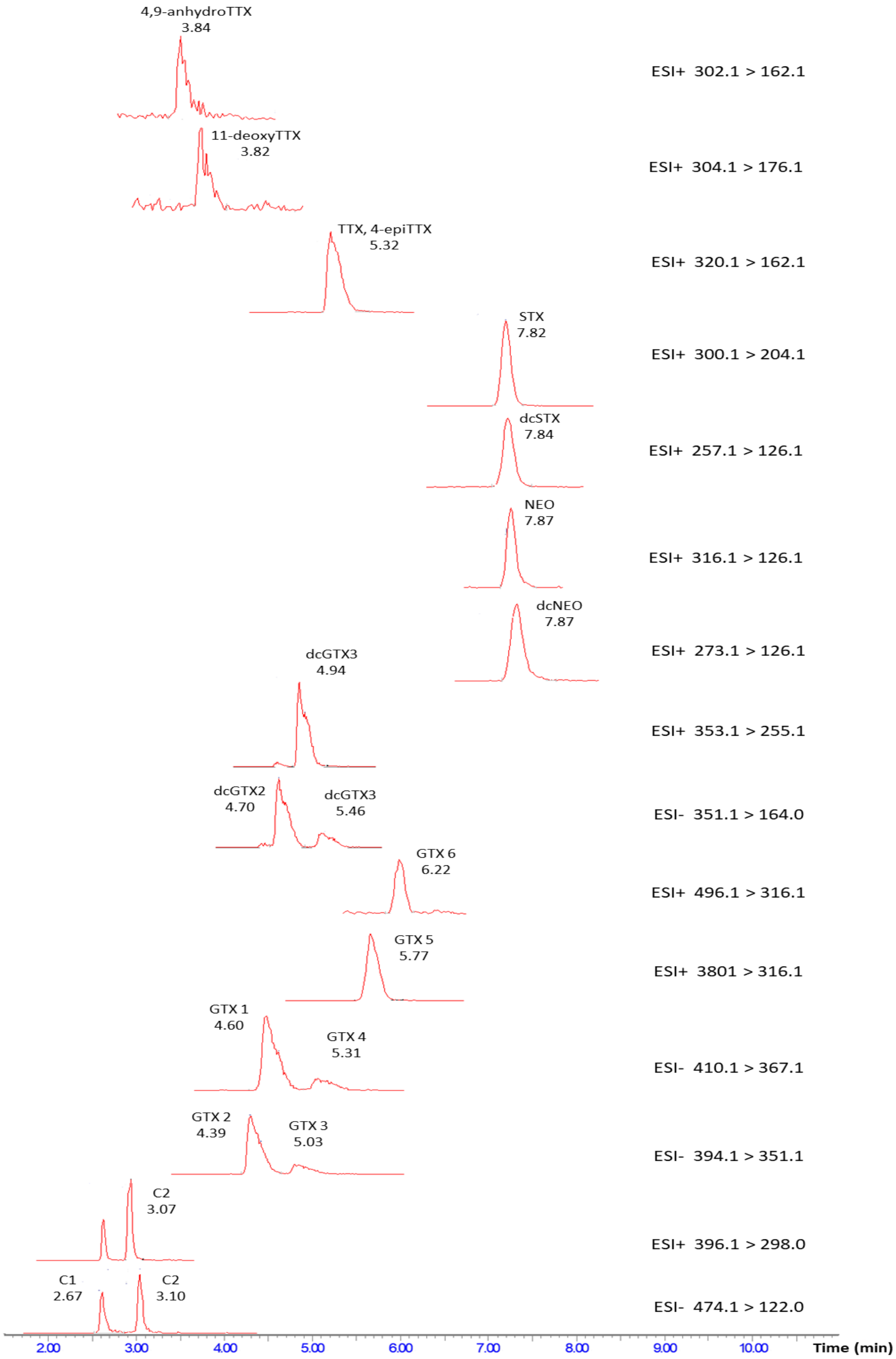

Figure S2. Full MRM transitions of each toxins at mid-level concentration spiked in blue mussel matrix.

Table S1. Concentrations of the calibration standard solutions.

Calibration Standard Solutions	Calibration standard concentration (nmol/L)																
	**dcGTX2**	**dcGTX3**	**dcSTX**	**GTX1**	**GTX4**	**GTX2**	**GTX3**	**NEO**	**STX**	**C1**	**C2**	**dcNEO**	**GTX5**	**GTX6**	**TTX**	**4,9-anhydroTTX**	**11-deoxyTTX**
	nmol/L																
S1	200.20	58.80	130.00	114.40	36.00	205.20	87.00	130.20	132.60	226.80	67.80	60.80	116.98	25.00	157.20	5.00	19.80
S2	100.10	29.40	65.00	57.20	18.00	102.60	43.50	65.10	66.30	113.40	33.90	30.40	58.49	12.50	78.60	2.50	9.90
S3	50.05	14.70	32.50	28.60	9.00	51.30	21.75	32.55	33.15	56.70	16.95	15.20	29.25	6.25	39.30	1.25	4.95
S4	25.03	7.35	16.25	14.30	4.50	25.65	10.88	16.28	16.58	28.35	8.48	7.60	14.62	3.13	19.65	0.63	2.48
S5	12.51	3.68	8.13	7.15	2.25	12.83	5.44	8.14	8.29	14.18	4.24	3.80	7.31	1.56	9.83	0.32	1.24
S6	6.26	1.84	4.06	3.58	1.13	6.41	2.72	4.07	4.14	7.09	2.12	1.90	3.66	0.78	4.91	0.16	0.62

Calibration Standard Solutions	Calibration standard concentration as equivalent concentration of saxitoxin (µg STX eq/kg)																
	**dcGTX2**	**dcGTX3**	**dcSTX**	**GTX1**	**GTX4**	**GTX2**	**GTX3**	**NEO**	**STX**	**C1**	**C2**	**dcNEO**	**GTX 5**	**GTX 6**	**TTX**	**4,9-anhydroTTX**	**11-deoxyTTX**
	µg STX eq/kg															µg TTX eq/kg	
S1	272.56	120.08	671.53	941.16	207.32	649.00	412.74	821.02	793.69	21.56	64.46	167.90	88.75	19.77	100.33	30.31	119.23
S2	136.28	60.04	335.77	470.58	103.66	324.50	206.37	410.51	396.85	10.78	32.23	83.95	44.38	9.88	50.16	15.16	59.62
S3	68.14	30.02	167.88	235.29	51.83	162.25	103.19	205.25	198.42	5.39	16.12	41.97	22.19	4.94	25.08	7.58	29.81
S4	34.07	15.01	83.94	117.64	25.91	81.13	51.59	102.63	99.21	2.70	8.06	20.99	11.09	2.47	12.54	3.79	14.90
S5	17.03	7.50	41.97	58.82	12.96	40.56	25.80	51.31	49.61	1.35	4.03	10.49	5.55	1.24	6.27	1.89	7.45
S6	8.52	3.75	20.99	29.41	6.48	20.28	12.90	25.66	24.80	0.67	2.01	5.25	2.77	0.62	3.14	0.95	3.73
